# The impact of COVID-19 on communicative accessibility and well-being in adults with hearing impairment: a survey study

**DOI:** 10.1186/s12889-023-15514-0

**Published:** 2023-04-05

**Authors:** Annemiek Hammer, Martine Coene

**Affiliations:** Faculty of Humanities, Applied Linguistics, Vrije Universiteit Amsterdam, Boelelaan 1105, 1081 HV Amsterdam, Netherlands

**Keywords:** Hearing impairment, Communication, Accessibility, Well-being, Resilience, COVID-19, Speech perception, Pandemic

## Abstract

**Background:**

COVID-19 measures, such as face masks, have clear consequences for the communicative accessibility of people with hearing impairment because they reduce speech perception. As communication is essential to participate in society, this might have impact on their mental well-being. This study was set out to investigate the impact of the COVID-19 measures on the communicative accessibility and well-being of adults with hearing impairment.

**Method:**

Two groups of adults took part in this study, with (*N* = 150) and without (*N* = 50) hearing loss. The participants answered statements on a five point Likert-scale. Statements regarding communicative accessibility involved speech perception abilities, behavioral changes and access to information. Well-being was measured at the overall level in daily community life and at work, and in particular also with respect to perceived stress. We asked participants with hearing impairment on their audiological needs during the pandemic.

**Results:**

Significant group differences were found on speech perception abilities due to COVID-19 measures. Behavioral changes were observed to compensate for the loss in speech perception. Hearing loss was associated with an increased request for repetition or for removal of the face mask. Using information technology (e.g. Zoom) or contacting colleagues did not pose any major problems for the hearing group, whereas participants with hearing loss gave mixed responses. A significant difference emerged between groups on well-being in daily life, but not on well-being at work or perceived stress.

**Conclusions:**

This study shows the detrimental effect of COVID-19 measures on the communicative accessibility of individuals with hearing loss. It also shows their resilience as only partial group differences were found on well-being. Protective factors are indicated, such as access to information and audiological care.

**Supplementary Information:**

The online version contains supplementary material available at 10.1186/s12889-023-15514-0.

## Background

The COVID-19 pandemic has made drastic changes in people’s individual and social life. To reduce potential airborne transmission of respiratory droplets, government officials mandated to wear face-masks and to keep social distance in public places. Some groups, however, might have been disproportionally affected by such COVID-19 precautions due to their disability [1].

Given that face masks have a detrimental effect on communication for people with hearing impairment, the COVID-19 precautions may be expected to have clear consequences for individuals with hearing impairment. Face masks cover 60 – 70% of the lower part of the face limiting the use of visual cues from lips and mouth to aid speech perception [2]. It has been shown that adults with hearing impairment rely more heavily on visual cues during speech recognition as compared to normal hearing adults [3, 4]. Indeed, adults with hearing loss reported to have become more aware of the extent to which they relied on visual cues during COVID-19 when face masks had become ubiquitous [5, 6]. Face masks act as a low-pass filter reducing sound levels by approximately 7 – 13 dB for higher frequencies (i.e. between 2 and 5 kHz). Furthermore, different types of face masks exhibit different acoustic effects for frequencies greater than 2 kHz [7, 8]. Higher frequency acoustic information is beneficial for speech understanding in particular when background noise is present [9]. In a speech recognition test, one-syllable words were offered in a masked and unmasked condition to normal hearing adults. It was found that speech intelligibility decreased with 12% to 16% in the masked condition as compared to the unmasked condition [10]. Speech perception has been shown to decrease even further for adults with hearing impairment [11]. For adults with hearing impairment, speech perception scores significantly decreased when sentences were offered in a masked condition (surgical mask) as compared to an unmasked condition. Interestingly, transparent face masks or face shields improved speech perception significantly over a masked condition for normal hearing adults [12] as well as for adults with hearing impairment [9]. This indicates that visual cues partly compensate for the acoustic impact of masks in speech perception. The precaution of physical distancing compounds further on the effects of wearing face masks, as sound intensity decreases as function of distance. This leads to an extra reduction of the audibility of the speech signal [13, 14].

The reduced communicative accessibility for adults with hearing loss during COVID-19 elicited negative emotions, such as anxiety, stress or isolation [5, 6]. Degree of hearing loss, as measured by self-reported hearing ability, affected the feeling of engagement in conversations with others who wore face masks and their levels of anxiety when they spoke to someone wearing a face mask [5, 6]. Adults with self-classified poor hearing ability were generally less communicatively engaged and reported higher levels of anxiety as compared to adults with better self-classified hearing [5, 15]. There were also behavioral changes reported in reaction to the communicative difficulties. This ranged from asking people to repeat or to remove their face mask to avoiding communication situations. A higher prevalence of symptoms of depression, anxiety, or other forms of psychological distress during the pandemic has been reported for the general public [16]. Given the fact that adults with hearing impairment are psychologically more vulnerable [17] and at risk for social isolation [18], it is likely that the impact of COVID-19 on their well-being goes beyond communicative accessibility only and affects their overall daily life.

Amongst the factors that might protect adults with hearing impairment against symptoms of psychological problems we find the access to updated and accurate COVID-19 related information from authorities and audiological care [15]. Up-to-date health information on COVID-19 at the initial stage of the outbreak was associated with lower levels of stress and anxiety in normal hearing adults [19]. It has been shown that many of the adults with hearing impairment do not listen to radio for updates about COVID-19, but find TV updates in general easy to follow [15]. During the pandemic, hearing aid services including hearing screening, audiological assessment or hearing aid programming have not been consistently accessible. Alqudah et al. [20] found that adults with hearing impairment faced hearing difficulties very frequently and experienced hearing-related problems such as tinnitus. Participants of this study emphasized the importance of tele-audiology. Yet, only a few audiologists make use of such remote audiology [21].

Not only health care services faced challenges in using communication technology, also working adults were required to use this technology when forced to work from home. Work from home is in general difficult for employees and might lead to become less engaged [22]. Adults with hearing impairment indicated that hearing was compromised in video calls, especially for those with moderate to severe hearing loss [15]. However, participants in this study also highlighted the fact that background noise was reduced when making calls from home. Therefore, the impact of COVID-19 measures on communicative accessibility and well-being might differ depending on the context, in daily life or at work.

### Study aim

The aim of this study is to investigate the impact of the COVID-19 measures on the communicative accessibility and well-being of adults with hearing impairment. Previous studies involving this target group did not include a control group. Yet, it is unclear if adults with hearing impairment are affected more seriously by the COVID-19 measures as compared to hearing controls. Therefore, in this study we address the question to what extent working adults with hearing impairment are affected by COVID-19 measures when compared to working adults with normal hearing on communicative accessibility and well-being. As COVID-19 measures place speech perception under pressure, we expect to find poorer outcomes for adults with hearing impairment on communicative accessibility. Based on previous studies regarding the impact of COVID-19 measures on adults with hearing impairment and given that fact that they are psychologically more vulnerable, we expected to find lower scores on well-being for the hearing impaired as compared to controls. When specified to the working context, hypotheses are less clear cut. Results between participants, regardless of hearing status, might vary depending on skills in using information technology and support by employers and colleagues. In addition, video calling also has benefits for the hearing impaired such as less hinder of noise. As such, we expected to find group differences on communicative accessibility and well-being in the context of daily life, but not when applied to the context of work.

## Method

This study was conducted in the first half of 2021 in The Netherlands and Belgium. The year started with a complete lock down followed by phased opening from February onwards. Between January and June the following COVID-19 measures were in force: wearing face masks and keep physical distance in public places, receiving a minimal number of guests at home and working from home. In January vaccination started and vaccines were given by birth year. Most working adults received their first vaccine by June. Ethical approval for this study was given by the VU-FGW Ethical Board on March 2021 (ETCO21.04).

### Participants

Two groups participated in this study. The first group (*N* = 50) were working adults with normal hearing (NH) who were between 22 – 64 years of age. The second group (*N* = 150) involved adults with hearing impairment (HI) between 23 – 64 years of age. Group details are presented in Table 1. Participants were placed in one of the groups based on self-reported hearing. For the adults in the HI-group, 97% responded to have diagnosed hearing loss and 3% responded they thought to have hearing loss but no diagnosis was given. Subsequently, they were asked to indicate the degree of hearing loss for each ear. For analysis, we only report their hearing loss for the best ear. Informed consent was obtained from all participants prior to participation in the study.

**Table 1:** Sample and group characteristics (HA = Hearing Aid, CI = Cochlear Implant)

*Characteristic*	*Normal Hearing*	*Hearing Impaired*
**Number of participants**	*50*	*150*
**Age in years, mean (SD)**	*39.6 (12.3)*	*45.5 (10.6)*
**Female, n (%)**	*34 (68.0)*	*122 (81.6)*
**Highest educational level, n (%)**		
< *bachelor level*	*7 (14.0)*	*55 (36.7)*
≥ *bachelor level*	*36 (72.0)*	*71 (47.3)*
*Unknown*	*7 (14.0)*	*24 (15.9)*
**Occupational sector, n (%)**		
*Healthcare and social care*	*8 (16.0)*	*46 (30.7)*
*Education*	*22 (44.0)*	*26 (17.3)*
*Trade and services*	*4 (8.0)*	*25 (16.7)*
*Other*	*10 (20.0)*	*24 (16.0)*
*Unknown*	*6 (12.0)*	*29 (19.3)*
**Degree of Hearing loss, n (%)**		
*Mild (26 – 40 dB)*		*11 (7.3)*
*Moderate (41 – 60 dB)*		*51 (34.0)*
*Severe (61 – 80 dB)*		*57 (38.0)*
*Deaf (> 80 dB)*		*16 (10.7)*
*Unknown*		*15 (10.0)*
**Type of Hearing devices, n (%)**		
*None*		*21 (14.0)*
*Bilateral/unilateral HA*		*100 (66.7)*
*Bilateral/unilateral CI*		*9 (6.0)*
*Bimodal (CI/HA)*		*7 (4.6)*
*Unknown*		*13 (8.7)*

### Survey

For the purpose of this study a survey was designed consisting of five sections (Additional file 1: Appendix A). The first section included questions to get more insight in the sample characteristics. A summary of findings is given in Table 1. In the following sections participants were asked to rate statements on a five point Likert scale from strongly disagree to strongly agree (or Not Applicable N/A). The second section involved statements regarding communicative accessibility and well-being in general (or daily life). Communicative accessibility was split up in several themes: speech perception abilities, behavioral changes in response to COVID-19 measures and access to information. The third section asked participants to indicate to what extent COVID-19 measures impacted on their working lives. The last section was only relevant for the HI group. They were asked about their access to and need of audiological care during the lock down.

### Procedure and data-analysis

Data were collected via Thesistools, an online survey program, in May and June 2021. The survey was spread via the personal network of researchers and Linked-In. Two audiological centers supported data collection. Participants visiting these centers received the survey-in-print or received an email with the survey link via email.

Accordingly, data from Thesistools were exported into IBM Statistics 25 [23]. Prior to analyses, responses of ‘N/A’ were excluded from all calculations. Therefore, the total N varies from item to item. Effective response rates for each item varied from 35/50 to 47/50 responses in the NH group and from 80/150 to 139/150 in the HI group. Cronbach’s Alpha was performed to measure if items consistently reflect the construct to be measured. The concept of *well-being* has been operationalized into three constructs: well-being in daily life, at work and perceived stress in daily life. The concept of *communicative accessibility* was split up in five constructs: speech perception, communicative behavior, communication at home and at work, and access to information. An alpha of >  = 0.70 was taken as indication of good reliability between items and around 0.60 as fair. These items were collapsed into a composite variable for each participants (summing the scores on items and dividing it by the number of items). Scores on positive formulated items were rotated. High composite scores indicate a higher degree of hindrance by COVID-19 measures.

Composite scores per construct were used to statistically test group differences. We used a one-way ANOVA with alpha 0.05, or if assumptions were violated, a Mann–Whitney U-test. In the case of an Cronbach’s alpha < 0.60 we conducted a Chi-Square test to assess the contrast between both groups on a 5 × 2 cross tabulation of response (five categories) and group (two categories). For items that were specific for the hearing impaired group we used a one sample Chi Square test.

## Results

### Well-being during COVID-19

**Fig. 1:**
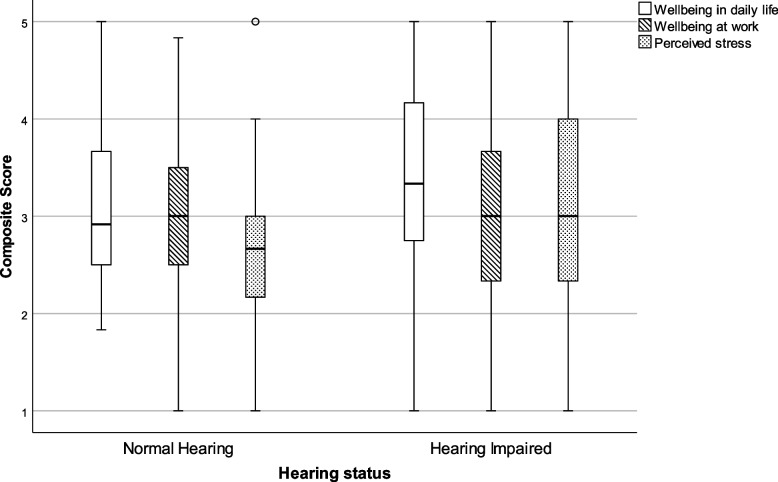
Box-and-whisker plots of the composite scores on well-being in daily life, at work and perceived stress. Boxes indicate the range of the central 50% of the data, with a central line marking the median value. Whiskers capture the range of the remaining data, with dots placed past the line edges indicating outliers

A total of 15 items evaluated the well-being of participants. Participants were asked to judge their well-being in daily life (six items, alpha 0.830) and at work (six items, alpha 0.766) as well as perceived stress in private life (three items, 0.592). Composite scores were calculated for both groups separately. As data were not normally distributed, the results are presented by means of 5 parameter statistics in Table 2 and visually presented by means of a Box-and-Whisker plot in Fig. 1.

**Table 2:** Composite scores for wellbeing in daily life and at work and for perceived stress

Well-being	Hearing status	N	Minimum	Lower quartile	Median	Upper quartile	Maximum	Statistical outcomes
**In daily life**	NH	46	1.8	2.5	2.9	3.7	5.0	U = 3748.0 *p* = .014
	HI	131	1.0	2.7	3.3	4.2	5.0	
**At work**	NH	29	1.0	2.4	3.0	3.6	4.8	U = 1046.0 *p* = .926
	HI	73	1.0	2.3	3.0	3.7	5.0	
**Perceived stress**	NH	36	1.0	2.1	2.7	3.0	5.0	U = 2144.5 *p* = .108
	HI	101	1.0	2.3	3.0	4.0	5.0	

*NH* Normal Hearing, *HI* Hearing Impaired

On well-being in daily life a small, but significant difference between groups was detected with a median score of 2.9 for normally hearing participants as compared to 3.3 for participants with a hearing impairment. No statistical difference was found between the groups on well-being at work (median score NH = 3.0 vs HI = 3.0), nor for perceived stress (median score NH = 2.7 vs HI = 3.0).

### Communicative accessibility during COVID-19

**Fig. 2:**
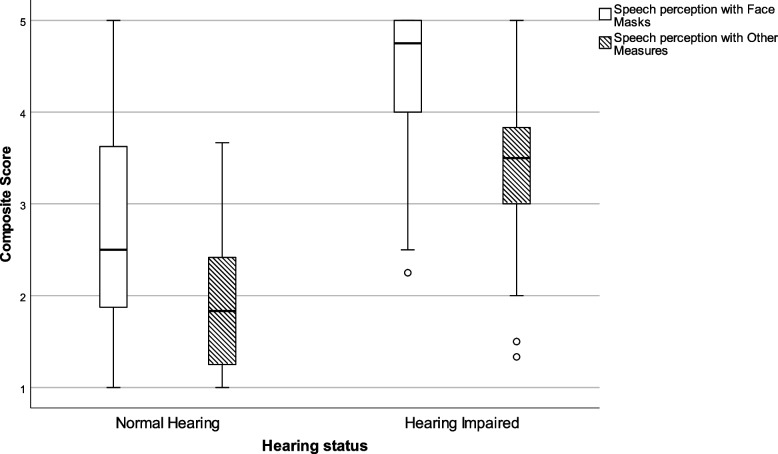
Box-and-whisker plots of the Composite scores on communicative accessibility with COVID-19 measures. Boxes indicate the range of the central 50% of the data, with a central line marking the median value. Whiskers capture the range of the remaining data, with dots placed past the line edges indicating outliers

There were nine items asking participants to judge the ease with which they were able to perceive speech when COVID-19 measures were present (alpha 0.893). One of the most salient COVID-19 measure was wearing face masks. As such, these items are plotted separately (four items, alpha 0.950) from other COVID-19 measures that might affect speech perception, e.g. plexiglass barriers and physical distancing (five items, alpha 0.760). As is shown in Table 3 and Fig. 2, clear differences emerge between groups. The HI group scores significantly higher (median = 4.8) as compared to the NH group (median = 2.5) for speech perception with face masks, as well as for speech perception with other COVID-19 measures (median NH = 1.7 against median HI = 3.2). Higher scores imply that participants with hearing impairment had more difficulty perceiving speech when face masks are worn, when spoken to at a larger distance than normal or when plastic barriers were involved.

**Table 3:** Composite scores for communicative accessibility in the presence of COVID-19 measures

Communicative accessibility	Hearing status	N	Minimum	Lower quartile	Median	Upper quartile	Maximum	Statistical outcomes
**Face Masks**	NH	40	1.0	1.8	2.5	3.7	5.0	U = 5087.0 *p* < .000
	HI	137	2.3	4.0	4.8	5.0	5.0	
**Other Measures**	NH	32	1.0	1.2	1.7	2.6	3.8	U = 3301.5 *p* < .001
	HI	117	1.4	2.8	3.2	3.6	5.0	

*NH* Normal Hearing, *HI* Hearing Impaired

**Fig. 3:**
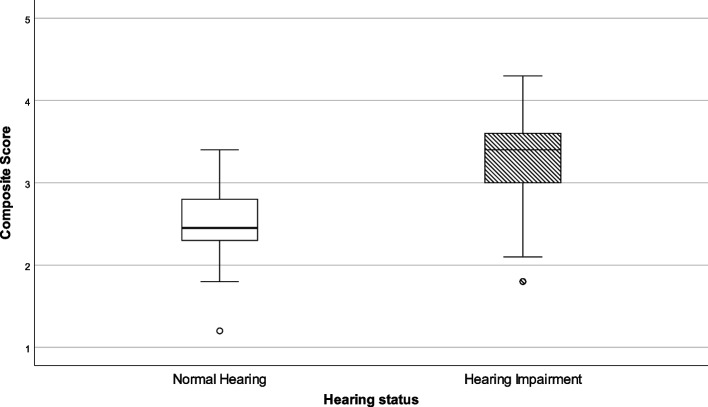
Box-and-whisker plots of the Composite scores on communicative behaviour using information technology. Boxes indicate the range of the central 50% of the data, with a central line marking the median value. Whiskers capture the range of the remaining data, with dots placed past the line edges indicating outliers

Communicative behavior was measured with 10 items. Four items (alpha 0.647) asked participants to judge their experience with video conferencing. Results of both groups are presented in Table 4 and in Fig. 3. A significant difference between groups was observed with a median score of 2.5 for NH participants against a median score of 3.4 for HI participants. A more detailed analysis revealed that participants in the NH group did not experience difficulty in perceiving speech or disentangle who is speaking in a video call whereas participants in the HI group gave mixed responses.

**Table 4:** Composite scores for communicative behaviour in the presence using information technology

Communicative behaviour	Hearing status	N	Minimum	Lower quartile	Median	Upper quartile	Maximum	Statistical outcomes
**Appreciation of information technology**	NH	34	1.2	2.3	2.5	2.8	3.4	U = 1335.0 *p* < .000
	HI	111	1.8	3.0	3.4	3.6	4.3	

*NH* Normal Hearing, *HI* Hearing Impaired

The other six items asked participants about their communicative behavior in response to reduced speech perception, for instance asking for repetition or avoiding communicative situations. Cronbach’s alpha was poor (0.459) and therefore we conducted a Chi square tests on each item individually. Three items revealed significant results, see Table 5. The majority of the participants in the HI group asked for repetition more often than NH participants. Around 50% of the participants in the HI group asked speakers to remove their face mask when talking or preferred communication with speakers they know. Such a pattern was not observed for the NH group.

**Table 5:** Results of items on communicative behavior in response to reduced speech perception

	Communicative behavior in response to speech perception	NH-group			HI-group		Statistical results	
	N (%)	Disagree	Neutral	Agree	Disagree	Neutral	Agree	
1	Due to corona measures, I have to ask for repetition more often	25 (65.7)	3 (7.9)	10 (26.3)	3 (2.2)	10 (7.2)	126 (90.7)	X^2^(4) = 103.53 *p* < .000
2	It is hard for me to keep in touch with family and friends during the lockdown	12 (27.9)	12 (27.9)	19 (44.2)	44 (31.8)	27 (19.6)	67 (48.5)	X^2^(4) = 5.64 *p* = .228
3	I easily dare to ask family or friends who wear a face mask to repeat if I don’t understand them	2 (4.8)	0 (0.0)	38 (95.0)	9 (6.8)	2 (1.5)	122 (91.8)	X^2^(4) = 6.60 *p* = .159
4	I easily dare to ask strangers who wear a face mask to repeat if I don’t understand them	2 (4.8)	4 (9.5)	36 (85.7)	18 (13.0)	16 (11.5)	105 (75.5)	X^2^(4) = 3.99 *p* = .407
5	I regularly ask if someone wants to take off his/her face mask when they speak to me	38 (92.7)	2 (4.9)	1 (2.4)	59 (42.4)	17 (12.2)	63 (43.4)	X^2^(4) = 37.51 *p* < .000
6	I prefer to communicate with acquaintances during the lockdown than with strangers	14 (35.0)	14 (35.0)	12 (30.0)	21 (15.5)	29 (21.3)	86 (63.3)	X^2^(4) = 20.45 *p* < .000

**Fig. 4:**
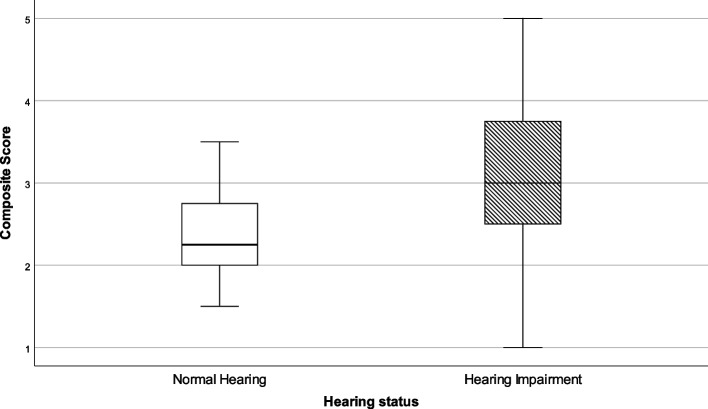
Box-and-whisker plots of the Composite scores on contact with colleagues. Boxes indicate the range of the central 50% of the data, with a central line marking the median value. Whiskers capture the range of the remaining data, with dots placed past the line edges indicating outliers

In the survey, four items questioned to what extent participants could get in contact and gather online with colleagues (alpha 0.659). Significant group differences were observed, as shown in Table 6 and Fig. 4. Participants in the NH group experienced no difficulty, whereas there was a large amount of variation in the outcomes of the participants in the HI group. Some participants had difficulty getting in touch with colleagues whereas others gave more neutral responses.

**Table 6:** Composite scores for contact with colleagues

	Hearing status	N	Minimum	Lower quartile	Median	Upper quartile	Maximum	Statistical outcomes
**Contact with colleagues**	NH	36	2.0	2.0	2.3	2.8	4.0	U = 900.0 *p* < .000
	HI	96	1.0	2.5	3.0	3.8	5.0	

*NH* Normal Hearing, *HI* Hearing Impaired

We asked participants in the HI group three specific questions related to their hearing loss. Approximately half of the participants indicated that colleagues were sensitive to their hearing impairment. The majority of the participants (around 70%) were more aware of their hearing problems since the lockdown. No clear results were found on how they work with interpreters. There were only a small number (*N* = 36) who answered this question. Results are placed in Additional file 2: Appendix B.

We included four items that evaluated to what extent communication with family changed in response to the COVID-19 measures. Reliability between items was too low (alpha 0.464) to calculate composite scores. Therefore, Chi square tests were conducted for each item.. No relation was found between group and responses, see Table 7. This indicates that hearing impairment did not affect communication at home during COVID-19.

**Table 7:** Statistical results of items on communication with family

	Communicative behavior at home	NH-group			HI-group		Statistical results	
	N (%)	Disagree	Neutral	Agree	Disagree	Neutral	Agree	
1	I’d rather leave things that require me to go outside to someone else	31 (77.5)	3 (7.5)	6 (15.0)	77 (57.5)	22 (16.4)	35 (26.1)	X^2^(4) = 7.89 *p* = .096
2	I find it difficult to keep in touch with family and friends	23 (57.5)	7 (17.5)	10 (25.0)	57 (42.6)	27 (20.1)	50 (37.7)	X^2^(4) = 6.56 *p* = .161
3	The corona measures have no effect on communicating with family members	11 (29.0)	7 (18.4)	20 (52.7)	40 (31.2)	12 (9.4)	76 (59.4)	X^2^(4) = 3.92 *p* = .417
4	I can easily ask family and friends for repetition if I don’t understand them	1 (2.9)	0 (0.0)	34 (97.1)	8 (6.0)	8 (6.0)	118 (88.0)	X^2^(4) = 5.67 *p* = .225

### Access to information during COVID-19

**Fig. 5:**
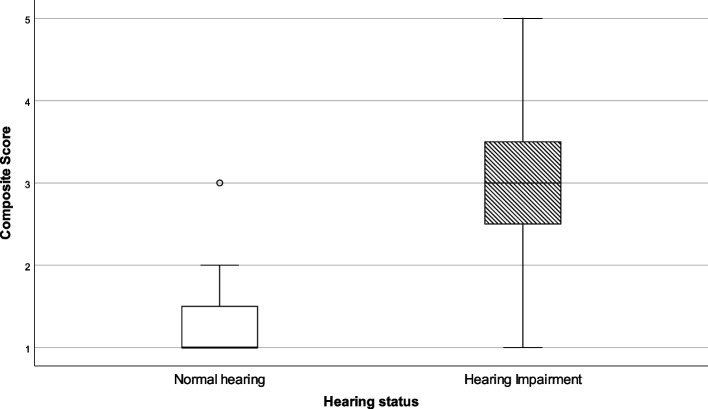
Box-and-whisker plots of the Composite scores on Access to Information. Boxes indicate the range of the central 50% of the data, with a central line marking the median value. Whiskers capture the range of the remaining data, with dots placed past the line edges indicating outliers

We included four items to evaluate to what extent participants had access to information regarding COVID-19 via television and radio (alpha 0.757). Composite scores for each group are presented in Table 8 and Fig. 5. This figure shows that participants with hearing impairment in general felt that they had less access to information (median score = 3.0) as compared to participants with normal hearing (median score = 3.0). This difference between the two groups was significant. The HI group were overall neutral to access to information.

**Table 8:** Composite scores for Access to Information

	Hearing status	N	Minimum	Lower quartile	Median	Upper quartile	Maximum	Statistical outcomes
Access to Information	NH	35	1.0	1.0	1.0	1.5	3.0	U = 249.0 *p* < .000
	HI	109	1.0	2.4	3.0	3.6	5.0	

*NH* Normal Hearing, *HI* Hearing Impaired

### Impact of hearing loss on speech perception abilities during COVID-19

The HI group responded more negative on items involving speech perception with face masks, physical distancing and plexiglass barriers. We performed a one-way ANOVA analysis to compare the effect of hearing loss on the constructs ‘speech perception with face masks’ and ‘speech perception with other COVID-19 measures’. Results (see Table 9) revealed that there was no significant effect of degree of hearing loss on both measures, i.e. little difference was found between the mean scores of participants with mild, moderate, severe hearing loss and deafness both with respect to the effect of face masks, and other COVID-19 measures.

**Table 9:** Composite scores for Speech Perception for subgroups of HI participants

Speech Perception	*Degree of HL*	*N*	*mean*	*SD*	*Lower bound 95% ci*	*Upper bound 95% ci*	*Statistical outcomes*
**Face Masks**	Mild	11	4.5	0.5	4.1	4.8	F(3, 125) = .161 *p* = .923
	Moderate	49	4.6	0.5	4.4	4.7	
	Severe	55	4.5	0.6	4.4	4.7	
	Deaf	15	4.6	0.7	4.3	5.0	
**Other Measures**	Mild	8	3.0	0.6	2.5	3.5	F (3, 106) = .621, *p* = .603
	Moderate	42	3.4	0.7	3.1	3.6	
	Severe	49	3.2	0.8	3.0	3.4	
	Deaf	12	3.3	0.5	3.0	3.6	

*HL* Hearing Loss, *Mild HL* 26–40 dB (HL), *Moderate HL* 41–60 dB (HL), *Severe HL* 61–90 dB (HL), Deaf > 80 dB (HL)

### Access to audiological care

We included six items asking the HI group to evaluate their audiological needs and access to audiology centers during COVID-19 (alpha 0.578). Results per item are presented in Table 10. Results indicate that most of the participants did not avoid audiology centers or were in need of more care during COVID-19. Also, the majority of the participants did not wear their hearing aids less often during the lockdown. With regard to the items on e-audiology, participants by and large responded that they had no experience with e-audiology. Reponses on the question if they would use e-audiology services are neither positive or negative.

**Table 10:** Outcomes on items evaluating access to audiological care

	Access to audiological care	Descriptives		
	N (%)	Disagree	Neutral	Agree
1	I avoid the audiology center during lock down	84 (68.5)	21 (16.9)	17 (14.5)
2	Due to the corona measures, I need more hearing care	68 (56.5)	26 (21.0)	26 (22.6)
3	Due to the lockdown, I wear my hearing aids less often than before the lockdown	95 (81.5)	6 (5.0)	15 (12.6)
4	Due to the corona measures, I need an adaptive program for my hearing aids	51 (44.7)	32 (28.9)	30 (26.3)
5	If my audiology center offered e-hearing care, I would use it	46 (38.0)	31 (25.6)	44 (36.4)
6	I already use e-hearing care	84 (87.6)	6 (6.3)	6 (6.3)

## Discussion

The COVID-19 pandemic has challenged the resilience of people with hearing impairment. This study investigated to what extent they felt pressured by the COVID-19 measures in their communication and well-being in daily life and at work. Obviously, the taken measures might also have consequences for people with normal hearing. As such, we included a control group. The group comparison sought to determine to what extent adults with hearing impairment are more negatively affected by the COVID-19 measures. We designed an extensive survey which was filled in by 150 working adults with hearing impairment and 50 with normal hearing.

We expected that adults with hearing impairment experienced reduced access to communication as COVID-19 measures such as wearing face masks and physical distancing limit the audibility of speech signals [7, 8, 11]. Results of this study show that adults with hearing impairment had significantly more trouble than normal hearing adults in perceiving speech of others when face masks were worn, when spoken to at a larger distance than normal or when plastic barriers were present. This finding is in accordance with other studies [5, 6, 15, 24]. In our study speech perception during COVID-19 was not more compromised for those with greater severity of hearing loss. This is in line with Naylor et al. [15] who found a widespread difficulty among their hearing impaired respondents on questions related to the understanding of speech when face masks are worn. Poon and Jenstad [6], however, showed that participants with greater severity of hearing loss reported significantly more often difficulties understanding others who wore face masks as compared to those with better hearing. The lack of significance in our study might be due to low samples sizes per hearing loss category. The study of Poon and Jenstad included four times more participants as our study and the study of Naylor et al. [15]. It might also be the case that communication in a face masked condition is mediated by an individual’s speech perception ability. Homans & Vroegop [11] found a larger impact on speech perception scores in a face masked condition for those participants who performed worse on a audiological speech perception task where words were offered in quiet at 65 dB SPL. More research is needed to give a clearer view on the potential effect of degree of hearing loss on speech perception in the particular context of speakers wearing face masks.

In response to reduced speech perception abilities, adults with hearing impairment asked for repetition more often or removal of the face masks irrespective of the speaker being known to them or not. A slight preference was observed for communication with acquaintances during the lockdown than with strangers in the hearing impaired group. This is in line with other studies in which a change of conversational tactics was found as well [6, 15].

Due to social distancing, most communication with family and friends, as well as with colleagues, took place by means of video calling. For adults with normal hearing, speech perception in video calls did not pose any major problems. Similarly, getting in contact with colleagues by telephone or using information technology was not difficult for them. However, on both composites, adults with hearing impairment gave more mixed results and significant group differences were found. This is in line with Naylor et al. [15] who also found mixed responses on questions related to enjoyment of video calls and speech perception in these calls. They found a slight tendency for those with worse hearing to respond more negatively as compared to those with better hearing.

Interestingly, around 70% of the adults with hearing impairment indicated that they were more aware of their hearing loss during the lock down. Naylor et al. [15] also found that those individuals with worse hearing thought more about their hearing loss during COVID-19. However, increased awareness about their hearing loss and the fact that they experienced reduced communicative accessibility due to COVID-19 measures did not lead to more negative outcomes on well-being as compared to adults without hearing difficulties. As such, our hypothesis was not borne out as we expected to find lower perceptions of well-being for participants with hearing loss.

The fact that the impact of COVID-19 measures on well-being were not more pronounced for those with hearing impairment, might be explained by the overall positive outcomes on protective factors. Communicative accessibility at home was not compromised for individuals with hearing impairment. Also, they had sufficient access to information on COVID-19. In addition, outcomes of this survey suggest that there were no significant changes in the need of and access to audiological care. This is in contrast with the results of Alqudah et al. [20], who found that the unavailability of essential audiological services negatively affected satisfaction of individuals with hearing impairment. Yet, there are marked differences between countries were studies were conducted. In Jordan hearing equipment manufacturing companies were closed making it difficult to repair hearing aids. This was not the case in The Netherlands. Our study shows that the majority of the individuals with hearing impairment do not have any experience yet with e-audiology. A third of them are willing to try these services when applicable. As such, the lock down opens up new avenues for audiological care services.

### Study limitations

The number of participants in this study is relatively small. It is regularly observed that there is large variation in outcomes between individuals with hearing impairment. This was also found in our study. As such, group means might not be applicable to all individuals with hearing impairment. We opted for non-parametric testing, because assumptions for parametric statistical testing were violated. The limitation of this statistic is that it cannot control for covariates, such as age. It could be that differences between groups in well-being are significant after correcting for age. Another limitation of this study was that, due to the temporariness of the lock down, the survey could not be tested on psychometric criteria such as validity and reliability. Not all items of the survey used in this study could be summarized in constructs. Using existing surveys on well-being and adding questions on communicative accessibility would have made the survey even more lengthier than it already was.

## Conclusion

This study has shown that COVID-19 measures decreased communicative accessibility for individuals with hearing impairment. In particular face masks had a detrimental effect on speech perception. Yet, participants with hearing loss were not more affected in their mental well-being as compared to adults without hearing difficulties. This can be explained in the context of protective factors such as sufficient access to information and audiological care. This study underlines the importance of studying potential risks in conjunction with protective factors for groups with disabilities as it sheds light on the resilience of the group under study. However, the results of this study clearly call for greater awareness of masks as barriers to communication for people with hearing impairment in policy. Promoting conversational tactics such as speaking more slowly, increasing speech volume and, when a safe distance can be maintained, removing the face mask when speaking may be expected to aid speech perception for the hearing impaired. Being patient and opening up the conversation with the hearing impaired individual on how to enhance communication be it face-to-face or when using information technology has the potential to overcome uncomfortable feelings (such as frustration or embarrassment) in people with hearing impairment. These recommendations build on the reciprocal nature of communication.

## Supplementary Information

**Additional file 1:** **Appendix A.** Survey.

**Additional file 2:** **Appendix B.** Results of items related to perception of hearing loss at work during COVID-19.

## Data Availability

The dataset used and analyzed during the present study are available from the corresponding author upon reasonable request.

## Abbreviations

HA: Hearing Aid
CI: Cochlear Implant
HI: Adults with Hearing Impairment
NH: Adults with Normal Hearing
