# Supplementary material for: The impact of COVID-19 on communicative accessibility and well-being in adults with hearing impairment: a survey study
Source: BMC Public Health. 2023 Apr 5;23:652. doi: 10.1186/s12889-023-15514-0 (PMC10073781; doi:10.1186/s12889-023-15514-0)
Supplement: Supplementary file 1 — Additional file 1: Appendix A. Survey. [file 12889_2023_15514_MOESM1_ESM.docx]

**Appendix A Survey**

Thank you for participating in this research! We designed a survey that measures your perception of communicative accessibility and well being during the COVID-19 pandemic. Filling out this survey will take 15 to 30 minutes. Please read the information letter first. If you agree to participate we ask you kindly to sign the informed consent form.

Survey ‘Fitting in during Covid-19’

1. I am [ ] Male [ ] Female [ ] Other

2. I am _________ years old

3. In which county of The Netherlands or Belgium do you live? _________

4. What is your native (or first) language?

*Multiple answers are possible.*

- Dutch
- Dutch Sign Language
- Flemish Sign Language
- Other: ­_________________________

5. What is your highest educational degree?

- Elementary education/ vocational eduction
- Bachelor
- Master or PhD
- Unknown

6. I am employed [ ] no [ ] yes, in the following sector:

- Health care and social care
- Trade and services
- ICT
- Justice and public administration
- Agriculture, nature, fishery
- Media and communication
- Education
- Engineering and construction

7. How do you follow information on COVID-19?

*Multiple answers are possible.*

- I watch the news on tv
- I read the news in the paper
- I read online news sites via apps
- I read the news on social media
- I listen to the news on the radio
- I hear the news from friends and family
- Other: ___________________________________

8. How would you judge your hearing?

- I have normal hearing
- I have diagnosed hearing loss
- I think I have hearing loss

*Questions related to hearing loss*

9. When was hearing loss diagnosed?

- Congenital hearing loss
- Acquired hearing loss

10. Please indicate the degree of hearing loss for your LEFT ear:

- Normal hearing (0-25 dB HL)
- Mild hearing loss (26-40 dB HL)
- Moderate hearing loss (41-60 dB HL)
- Severe hearing loss (61-80 dB HL)
- Deaf (>80 dB HL)

11. Please indicate the degree of hearing loss for your RIGHT ear:

- Normal hearing (0-25 dB HL)
- Mild hearing loss (26-40 dB HL)
- Moderate hearing loss (41-60 dB HL)
- Severe hearing loss (61-80 dB HL)
- Deaf (>80 dB HL)

12. Are you wearing rehabilitation devices for your hearing loss?

*Multiple answers are possible.*

- No, I do not wear rehabilitation devices.
- Yes, a hearing aid in my left ear.
- Yes, a hearing aid in my right ear.
- Yes, a cochlear implant in my left ear.
- Yes, a cochlear implant in my right ear.

13. How many hours a day on average do you use your rehabilitation devices?

- 0 to 5 hours a day
- 5 to 10 hours a day
- 10 to 15 hours a day
- 15 to 20 hours a day
- 20 to 24 hours a day

The survey continues on the next page.

You will see statements on various topics. For each statement, you may indicate to what extent you agree or disagree with the statement. We use a five point Likert scale. If a statement does not apply to you, you may select ‘not applicable’.

14. Statements on well being in daily life

|  | Strongly disagree | Disagree | Neutral | Agree | Strongly agree | NA |
| --- | --- | --- | --- | --- | --- | --- |
| During the lockdown I feel as happy as before | 1 | 2 | 3 | 4 | 5 | 0 |
| Due to the lockdown I miss fun around me | 1 | 2 | 3 | 4 | 5 | 0 |
| Due to the lockdown I experience negative thoughts more often | 1 | 2 | 3 | 4 | 5 | 0 |
| The lockdown makes me feel more excluded than before | 1 | 2 | 3 | 4 | 5 | 0 |
| Since the lockdown I’m more frustrated than before | 1 | 2 | 3 | 4 | 5 | 0 |
| Since the lockdown I am less tired than before | 1 | 2 | 3 | 4 | 5 | 0 |

15. Statements on well being at work

|  | Strongly disagree | Disagree | Neutral | Agree | Strongly agree | NA |
| --- | --- | --- | --- | --- | --- | --- |
| Working from home makes it difficult to do my work. | 1 | 2 | 3 | 4 | 5 | 0 |
| Before lock down, I liked my job more. | 1 | 2 | 3 | 4 | 5 | 0 |
| Due to corona measures, I am less able to do my job well. | 1 | 2 | 3 | 4 | 5 | 0 |
| After a day of work I am more tired than before the lock down. | 1 | 2 | 3 | 4 | 5 | 0 |
| Contact with colleagues has diminished due to the lock down. | 1 | 2 | 3 | 4 | 5 | 0 |
| Due to the lock down, I often think about looking for another job. | 1 | 2 | 3 | 4 | 5 | 0 |

16. Statements on perceived stress

|  | Strongly disagree | Disagree | Neutral | Agree | Strongly agree | NA |
| --- | --- | --- | --- | --- | --- | --- |
| The combination of work, family and private life feels the same as before the lock down. | 1 | 2 | 3 | 4 | 5 | 0 |
| During the lock down it often feels like it is harder to keep it all in balance. | 1 | 2 | 3 | 4 | 5 | 0 |
| Since the lock down I have less time for myself. | 1 | 2 | 3 | 4 | 5 | 0 |

17. Statements on communicative accessibility – speech perception abilities

|  | Strongly disagree | Disagree | Neutral | Agree | Strongly agree | NA |
| --- | --- | --- | --- | --- | --- | --- |
| With a face mask I can't hear what the other is saying. | 1 | 2 | 3 | 4 | 5 | 0 |
| With a face mask I don’t understand the other well. | 1 | 2 | 3 | 4 | 5 | 0 |
| Because of the face mask I don’t see the mouth, so I can’t understand the other. | 1 | 2 | 3 | 4 | 5 | 0 |
| With the face mask, the voice is not loud enough for me. | 1 | 2 | 3 | 4 | 5 | 0 |
| I can understand people who wear a transparent face shield or a transparent face mask well. | 1 | 2 | 3 | 4 | 5 | 0 |
| When people without a face masks talk to me at a distance of 1.5 meters, I can understand them well. | 1 | 2 | 3 | 4 | 5 | 0 |
| When plexiglass is present, the voice is not loud enough for me. | 1 | 2 | 3 | 4 | 5 | 0 |
| Because of the reflection in the plexiglass, I don’t see the other enough when he/she speaks. | 1 | 2 | 3 | 4 | 5 | 0 |
| With plexiglass I tend to push it aside. | 1 | 2 | 3 | 4 | 5 | 0 |

18. Communicative behavior

|  | Strongly disagree | Disagree | Neutral | Agree | Strongly agree | NA |
| --- | --- | --- | --- | --- | --- | --- |
| Due to corona measures, I have to ask for repetition more often. | 1 | 2 | 3 | 4 | 5 | 0 |
| It is hard for me to keep in touch with family and friends during the lockdown | 1 | 2 | 3 | 4 | 5 | 0 |
| I easily dare to ask family or friends who wear a face mask to repeat if I don’t understand them. | 1 | 2 | 3 | 4 | 5 | 0 |
| I easily dare to ask strangers who wear a face mask to repeat if I don’t understand them. | 1 | 2 | 3 | 4 | 5 | 0 |
| I regularly ask if someone wants to take off his/her face mask when they speak to me. | 1 | 2 | 3 | 4 | 5 | 0 |
| I prefer to communicate with acquaintances during the lockdown than with strangers. | 1 | 2 | 3 | 4 | 5 | 0 |
| I now use video calling more than before the lockdown. | 1 | 2 | 3 | 4 | 5 | 0 |
| When we have video calls with more than two people at the same time, I can clearly distinguish who is saying what. | 1 | 2 | 3 | 4 | 5 | 0 |
| It is more difficult to understand people during video calls than during a conversation in the same room. | 1 | 2 | 3 | 4 | 5 | 0 |
| I appreciate video calling more than calling by telephone. | 1 | 2 | 3 | 4 | 5 | 0 |

19. Communication at work

|  | Strongly disagree | Disagree | Neutral | Agree | Strongly agree | NA |
| --- | --- | --- | --- | --- | --- | --- |
| If I video call with more than two colleagues, it is difficult for me to follow the conversation. | 1 | 2 | 3 | 4 | 5 | 0 |
| I find it difficult to contact a colleague by telephone. | 1 | 2 | 3 | 4 | 5 | 0 |
| I find meetings via zoom, facetime, teams, etc. more pleasant than on location. | 1 | 2 | 3 | 4 | 5 | 0 |
| I easily contact colleagues when I need them. | 1 | 2 | 3 | 4 | 5 | 0 |
| My colleagues take my hearing impairment into account. | 1 | 2 | 3 | 4 | 5 | 0 |
| I am more aware of my hearing impairment since the lockdown. | 1 | 2 | 3 | 4 | 5 | 0 |
| I can easily work in online with an interpreter. | 1 | 2 | 3 | 4 | 5 | 0 |

20. Communication at home

|  | Strongly disagree | Disagree | Neutral | Agree | Strongly agree | NA |
| --- | --- | --- | --- | --- | --- | --- |
| I’d rather leave things that require me to go outside to someone else. | 1 | 2 | 3 | 4 | 5 | 0 |
| I find it difficult to keep in touch with family and friends. | 1 | 2 | 3 | 4 | 5 | 0 |
| The corona measures have no effect on communicating with family members. | 1 | 2 | 3 | 4 | 5 | 0 |
| I can easily ask family and friends for repetition if I don’t understand them. | 1 | 2 | 3 | 4 | 5 | 0 |

21. Access to information

|  | Strongly disagree | Disagree | Neutral | Agree | Strongly agree | NA |
| --- | --- | --- | --- | --- | --- | --- |
| Television updates about corona are easy for me to follow. | 1 | 2 | 3 | 4 | 5 | 0 |
| Radio updates about corona are easy for me to follow. | 1 | 2 | 3 | 4 | 5 | 0 |
| I find the language about corona, that is used in the news, difficult to understand. | 1 | 2 | 3 | 4 | 5 | 0 |
| I need subtitles during press conferences about corona on television. | 1 | 2 | 3 | 4 | 5 | 0 |

22. Access to audiological care

|  | strongly disagree | disagree | neutral | agree | strongly agree | NA |
| --- | --- | --- | --- | --- | --- | --- |
| I avoid the audiology center during lock down. | 1 | 2 | 3 | 4 | 5 | 0 |
| Due to the corona measures, I need more hearing care. | 1 | 2 | 3 | 4 | 5 | 0 |
| Due to the lockdown, I wear my hearing aids less often than before the lockdown. | 1 | 2 | 3 | 4 | 5 | 0 |
| Due to the corona measures, I need an adaptive program for my hearing aids. | 1 | 2 | 3 | 4 | 5 | 0 |
| If my audiology center offered e-hearing care, I would use it. | 1 | 2 | 3 | 4 | 5 | 0 |
| I already use e-hearing care. | 1 | 2 | 3 | 4 | 5 | 0 |

This is the end of the survey. Thank you for your participation!
