# Supplementary material for: The impact of COVID-19 on communicative accessibility and well-being in adults with hearing impairment: a survey study
Source: BMC Public Health. 2023 Apr 5;23:652. doi: 10.1186/s12889-023-15514-0 (PMC10073781; doi:10.1186/s12889-023-15514-0)
Supplement: Supplementary file 2 — Additional file 2: Appendix B. Results of items related to perception of hearing loss at work during COVID-19. [file 12889_2023_15514_MOESM2_ESM.docx]

Appendix B

Results of items related to perception of hearing loss at work during COVID-19.

|  | Communication at work | HI-group | | | | | Statistical results |
| --- | --- | --- | --- | --- | --- | --- | --- |
|  | N (%) | strongly  disagree | disagree | neutral | agree | strongly  agree |  |
| 1 | My colleagues take my hearing impairment into account. | 11  (9.8) | 16 (14.3) | 34  (30.4) | 30  (26.8) | 20  (18.8) | X^2^(4) =16.07 p=.003* |
| 2 | I am more aware of my hearing impairment since the lockdown. | 17  (13.7) | 8  (6.5) | 13  (19.5) | 22  (17.7) | 64  (51.6) | X^2^(4) =81.73  P<.000* |
| 3 | I can easily work in online meetings with the interpreter. | 9  (25.0) | 3  (8.3) | 10  (27.8) | 6  (16.7) | 8  (22.2) | X^2^(4) =4.28  p=.396 |
